# COOPERATIVE-PFA: A Three-Arm Randomized Controlled Trial

**DOI:** 10.1161/CIRCULATIONAHA.125.074427

**Published:** 2025-04-27

**Authors:** Veronika Sochorová, Veronika Kunštátová, Pavel Osmančík, František Duška, Dalibor Heřman, Petr Waldauf, Lukáš Povišer, Jakub Karch, Lucie Znojilová, Věra Filipcová, Jana Hozmanová, Jana Veselá, Marek Hozman

**Affiliations:** 1 Cardiocenter, Third Faculty of Medicine, Charles University Prague and University Hospital Kralovske Vinohrady, Prague, Czech Republic (P.O., D.H., L.P., J.K., L.Z., V.F., J.H., J.V., M.H.).; 2 Department of Anesthesia and Intensive Care Medicine, Third Faculty of Medicine, Charles University Prague and University Hospital Kralovske Vinohrady, Prague, Czech Republic (V.S., V.K., F.D., P.W.).

**Keywords:** atrial fibrillation, catheter ablation, deep sedation, general anesthesia

## Abstract

**BACKGROUND:**

Deep analgosedation (DAS) or general anesthesia is mandatory for pulsed-field ablation of atrial fibrillation. In contrast to DAS, general anesthesia (conventional or total intravenous anesthesia [TIVA]) requires airway management. To find the optimal sedation regimen, this study compared ketamine-remimazolam DAS and propofol-opioid TIVA with propofol-opioid DAS, focusing on sedation-related adverse events.

**METHODS:**

Patients indicated for atrial fibrillation catheter ablation were randomly assigned at a 1:1:1 ratio to: (1) DAS using intermittent propofol-opioid boluses (arm P), (2) continuous remimazolam-ketamine DAS (arm R), or (3) continuous propofol-opioid TIVA with secured airway (arm TIVA). Catheter ablation was performed using the FARAPULSE system (Boston Scientific, MA). The major exclusion criterion was obstructive sleep apnea syndrome. The primary end point was defined as a composite of hypoxemia, hypotensive, or hypertensive events requiring intervention or leading to procedure discontinuation. Secondary end points included hemodynamic instability events, procedure time, serious adverse events, and patient satisfaction.

**RESULTS:**

One-hundred twenty-seven patients (mean age 62.9±10.3 years, 35.1% women, 47.2% with paroxysmal atrial fibrillation) were enrolled and randomized to the P (n=42), R (n=43), or TIVA (n=42) arms. The primary end point occurred in 85.7% of P patients, 27.9% of R patients, and 66.7% of TIVA patients (*P*<0.001), driven by hypoxemia in the P arm (100% of patients with the primary end point) and by hypotension in the TIVA arm (100%). The R arm showed a similar distribution of hypoxemia (50%) and hypotensive (66.7%) events. No differences were observed in mean procedural time, rate of serious adverse events, and assessment of patient satisfaction.

**CONCLUSIONS:**

In pulsed-field ablation procedures for atrial fibrillation, remimazolam-ketamine DAS was superior to propofol-opioid regimens (either boluses or continuous) and had the lowest risk of hypoxemia and hypotensive events. More than 80% of patients undergoing conventional propofol-opioid analgosedation experienced hypoxemia.

**REGISTRATION:**

URL: https://www.clinicaltrials.gov; Unique identifier: NCT06013345.

**Clinical Perspective**

**What Is New?**

- Our findings offer a new, safe deep analgosedation protocol suitable for pulsed-field ablation procedures in routine clinical practice.

**What Are the Clinical Implications?**

- The depth of analgosedation required and the rate of adverse events indicate that a dedicated sedation/anesthesia provider is required for the safety of the pulsed-field ablation procedure regardless of the type of sedation/anesthesia chosen.
- To assess its broader applicability, the remimazolam-ketamine deep analgosedation protocol should be evaluated in other cardiology interventions.

**Editorial, see p 160**

Catheter ablation represents the most effective treatment for drug-refractory symptomatic atrial fibrillation (AF).^1,2^ A novel technology, pulsed-field ablation (PFA), has recently entered clinical practice. In comparison to conventional thermal ablation techniques, such as radiofrequency or cryoablation, PFA induces tissue-selective lesions via non-thermal mechanisms through irreversible electroporation. Of note, PFA is faster and poses a lower risk of collateral tissue damage, such as an atrio-esophageal fistula or phrenic nerve palsy.^3,4^ Nevertheless, pulse applications, in particular using the unipolar pulse setting, cause significantly more pain and irritation of surrounding neuromuscular structures compared to traditional radiofrequency ablation. In turn, questions remain about how to make the PFA procedure tolerable, safe for patients, and convenient for physicians. In practice, this means providing an appropriate depth of sedation and analgesia without overuse of anesthetics and without unnecessarily using invasive techniques and scarce resources, such as airway-trained healthcare personnel. The depth of medical sedation is a continuum that ranges from mild sedation with anxiolysis through conscious and deep sedation to, finally, general anesthesia (GA). The principal difference between sedation and GA is the ability of patients to control their airway, and the main risk of deep sedation is the loss of airway control and subsequent hypoxemia. See Figure S1 for more details and infographics. Total intravenous anesthesia (TIVA) is a GA subgroup that exclusively uses intravenous anesthetics, avoiding the need for volatile agents. The broader term “analgosedation” refers to the use of a potent analgesic agent added to sedation regardless of the depth of sedation.

The first PFA procedures were performed using GA with orotracheal intubation and myorelaxation because the unipolar pulse setting caused severe fasciculations.^5,6^ Later, with the introduction of the bipolar pulse configuration, GA was no longer mandatory, and most centers reverted to the practice commonly used for RFA; ie, to perform PFA using bolus-based sedation without securing the airway.^6,7^

Because the doses required for PFA are much higher, we hypothesized that bolus-based sedation is associated with risks such as hypoxemia and loss of airway. Considering the benefits of continuous sedation^8^ and a favorable profile of ketamine in PFA procedures,^9^ we propose an alternative deep analgosedation (DAS) protocol using ketamine combined with continuous remimazolam, a benzodiazepine with an extremely short half-life. We hypothesize that this new regimen could offer a better safety profile in patients undergoing PFA without a secured airway. In this trial, we compare traditional, bolus-based sedation with the new regimen and TIVA with secured airways.

The objectives of the presented study were: (1) to evaluate whether optimized continuous DAS using remimazolam and ketamine or a propofol-based TIVA was associated with fewer clinically relevant adverse events compared with conventional DAS based on intermittent propofol boluses, and (2) to systematically assess the incidence of adverse events (ie, hypotension, hypertension, and hypoxemia), the depth of sedation (bispectral index [BIS] monitoring), and patient satisfaction across all 3 aforementioned regimens.

## METHODS

### Trial Design, Ethics, and Registration

This study was a prospective, randomized, parallel, single-blinded (subject-blinded), and single-center trial. The study was approved by the local ethics committee of the Kralovske Vinohrady University Hospital on September 6, 2023 (document number EK-VP/45/0/2023). The study was registered before the enrollment phase (https://clinicaltrials.gov; Unique identifier: NCT06013345). Informed consent was obtained from all study participants. The data that support the findings of this study are available from the corresponding author upon reasonable request.

### Study Subjects

We included patients ≥18 years of age with an indication for catheter ablation of AF (paroxysmal or nonparoxysmal) and who gave informed consent.

The main exclusion criteria were grade III to IV heart failure (New York Heart Association), significant valvopathy, and obstructive sleep apnea syndrome (on therapy or with an apnea-hypopnea index ≥30, measured using an ApneaLink device [ResMed, CA]). For the complete list of the exclusion criteria, see the Supplemental Appendix.

The night before the procedure, patients were admitted to the hospital and screened using an ApneaLink device. Subjects with an apnea-hypopnea index ≥30 were excluded and referred to a sleep laboratory.

### Study Procedures

All potentially eligible patients were invited to participate in the study and were admitted one day before the procedure to undergo a sleep study. According to local policy, patients fasted overnight, with clear fluids permitted until 2 hours before the procedure. The following morning, patients were randomly assigned at a 1:1:1 ratio to 1 of 3 groups: (1) conventional propofol DAS (arm P), (2) optimized continuous intravenous DAS based on remimazolam and ketamine (arm R), or (3) propofol-based TIVA with a secured airway (arm TIVA).

#### Ablation Procedure

Ablation procedures were performed using the FARAPULSE Pulsed Field Ablation System (Boston Scientific, MA) and the current guidelines at that time. Intracardiac echo guidance was used in all procedures. A wire-guided pentaspline ablation catheter was placed in the pulmonary vein ostia, where pulsed-field energy was used to create pulmonary vein isolation (PVI; 4 applications in the “basket” and 4 applications in the “flower” configuration with one-eighth-turn rotation between each application; ie, a minimum of 8 applications per vein). In nonparoxysmal AF cases, posterior wall and mitral isthmus ablation were performed in addition to PVI. If indicated, cavotricuspid isthmus ablation was performed (either by PFA or radiofrequency). Detailed procedural steps, including catheter techniques and anticoagulation management, are provided in the Supplemental Appendix.

#### Perioperative Monitoring

Standard 3-lead ECG, pulse oximetry, capnography, and invasive blood pressure monitoring were performed continuously during the procedure, using MetaVision software (IMDsoft, Tel Aviv, Israel). Sedation depth (arm P and TIVA) was measured using BIS monitoring (Covidien BIS Vista system, Medtronic, Ireland). An anesthesiologist and anesthesia assistant were present throughout every procedure and were blinded to BIS values. All DAS patients (ie, arm P and arm R) received oxygen via face mask at 5 L/min; capnography was used for early detection of apnea.

#### Study Arms and Interventions

Sedation for all patients was initiated during site disinfection and deepened 2 to 5 minutes before the beginning of the ablation phase (ie, after the transseptal puncture). An arterial blood gas sample was taken after the last ablation. For a detailed step-by-step description of anesthetic management in each arm, see Table S1.

In brief, patients in arm P received an initial bolus of intravenous midazolam, and the ablation phase was managed with 5 to 10 µg of sufentanil and a loading dose of propofol (0.8 to 1.0 mg/kg). An additional 0.5 mg/kg of propofol boluses were administered as needed during the procedure. If analgosedation was deemed inadequate, then additional boluses of midazolam or sufentanil were given. Sedation in arm R began with a loading dose of intravenous remimazolam, followed by continuous infusion of 0.5 mg/kg per hour, based on the patient’s ideal body weight. Before the ablation phase, a ketamine bolus of 1 mg/kg ideal body weight was administered. If necessary, then one additional bolus of ketamine (0.5 mg/kg ideal body weight) or 2.5 mg boluses of remimazolam (with no limit on the number of doses) could be administered. If analgosedation was inadequate, then a bolus of sufentanil was administered.

Patients randomized to the TIVA arm were initially sedated with a slow propofol infusion and a sufentanil bolus. GA was induced after the transseptal puncture using a bolus of sufentanil and propofol administered via a target-controlled infusion system (PK model Schnider, Perfusor Space, B. Braun, Germany). The target plasma concentration was set at 5 to 8 µg/mL for induction and adjusted to 3 to –6 µg/mL for maintenance of anesthesia. After administration of a reduced dose of rocuronium (0.2–0.4 mg/kg ideal body weight) to facilitate mechanical ventilation, the airway was secured with an iGel laryngeal mask airway (LMA). The ventilator was set to fraction of inspired oxygen 0.3 to 0.4, and minute ventilation was tailored to maintain an end-tidal carbon dioxide (EtCO_2_) of 30 to 40 mm Hg. Target-controlled infusion could be adjusted as necessary, and the infusion was discontinued after the last ablation pulse. The LMA was removed upon return to consciousness. For a step-by-step description of each arm, see the Supplemental Appendix.

### Outcome Assessment

The primary end point was a composite of hypoxemia, hypotensive, or hypertensive events requiring intervention (such as increased fraction of inspired oxygen, airway management, or drug administration) or resulting in procedure discontinuation.

Secondary outcomes included the following measures:

1. Total number of hemodynamic instability events per patient (hypoxemia, hypotension, and hypertension as defined above), with each continuous instability of 5 minutes counted as a new event and any instability persisting despite intervention also counted as a new event.
2. Total number of hemodynamic instability events per patient lasting >60 seconds, defined as hypoxemia (oxygen saturation <85%), hypotension (systolic blood pressure <85 mm Hg), or hypertension (systolic blood pressure >200 mm Hg) irrespective of the need for intervention.
3. Total number of interventions per patient (including airway maneuvers, use of nasopharyngeal airway, LMA, or orotracheal intubation; increased fraction of inspired oxygen; and use of vasoactive or antihypertensive drugs).
4. Total procedure time (skin to skin; ie, skin opening through skin closing).
5. Minimum BIS value recorded. Note: the BIS is a monitoring tool that measures the depth of sedation or anesthesia by analyzing brain wave activity, providing a numeric scale where lower values indicate deeper sedation or anesthesia.
6. Procedural sedation quality (measured using the PROcedural Sedation Assessment Survey 12 to 24 hours after the procedure).^10^
7. Difficulty of sedation score (1–10 scale: 1 = easy, 10 = difficult).
8. Operating physician satisfaction score (1–10 scale: 1 = satisfied, 10 = not satisfied).
9. Total number of serious adverse events. For the full list, see the Supplemental Appendix.
10. Post-procedural arterial PCO_2_.

### Statistical Considerations

The minimal sample size was estimated as follows. a mean incidence of propofol-related hypotension of 25% was calculated based on the data from 6 previously published trials.^11–16^ Similarly, the risk of hypoxemia related to propofol was calculated to have a mean value of 29% based on 3 previously conducted studies reporting the incidence of hypoxemia requiring intervention.^17–19^ Therefore, we anticipated the primary end point rate in the P arm to be 60% and in the TIVA arm to be 30% (lower in TIVA because of the absence of hypoxemia risk with a secured airway). Because we found no relevant data describing adverse events related to combining ketamine and remimazolam, we conducted a pilot study that found a 10% incidence of hypotension/hypoxemia requiring intervention (Hozman et al, Poster session, HRS Conference 2024, Boston, MA). However, in the power calculation, we used the anticipated primary end point rate of 30% for the R arm, as the primary end point in this study included more components. Based on our calculation, a sample of 126 patients had 80% power to detect a 5% 2-tailed significance level comparing TIVA with P and R with P. The power calculation did not include comparing R with TIVA.

Continuous data were reported as mean and SD, median and interquartile range (IQR) for nonparametric data, and counts and percentages for categorical data.

We used the χ^2^ or Fisher exact test for testing binary variables, followed by a subsequent pairwise proportion test adjusted for multiple comparisons using the Holm correction. To assess normally distributed data, we used ANOVA followed by the Tukey test. In data with a skewed distribution, the Kruskal-Wallis test was used, followed by a subsequent pairwise Wilcoxon rank sum test adjusted using the Holm correction for multiple comparisons. If only 2 independent means were compared, then we used the *t* test or Wilcoxon rank-sum test, depending on data distribution. All tests were 2 tailed, and *P*<0.05 indicated statistical significance.

All analyses were performed using R Statistical Software (v4.3.3, The R Foundation for Statistical Computing, 2024).

### Randomization, Masking, and Data Management

An electronic case report form was custom developed in Oracle to facilitate randomization and securely store data in a pseudonymized format. Permuted block randomization (block sizes of 3, 6, and 9) was employed, with stratification based on key characteristics that could affect the length of the procedure and the number of pulsed field applications: (1) PVI only versus PVI plus posterior wall or mitral isthmus ablation, and (2) cavotricuspid isthmus ablation performed or not performed. Treatment group allocation was not disclosed to the study participants. The patients were enrolled and assigned to the treatment group by one of the operating physicians just before the procedure in the electrophysiology laboratory. The operating physicians were not blinded to study arm allocation.

## RESULTS

### Patients and Procedures

**Figure 1.**
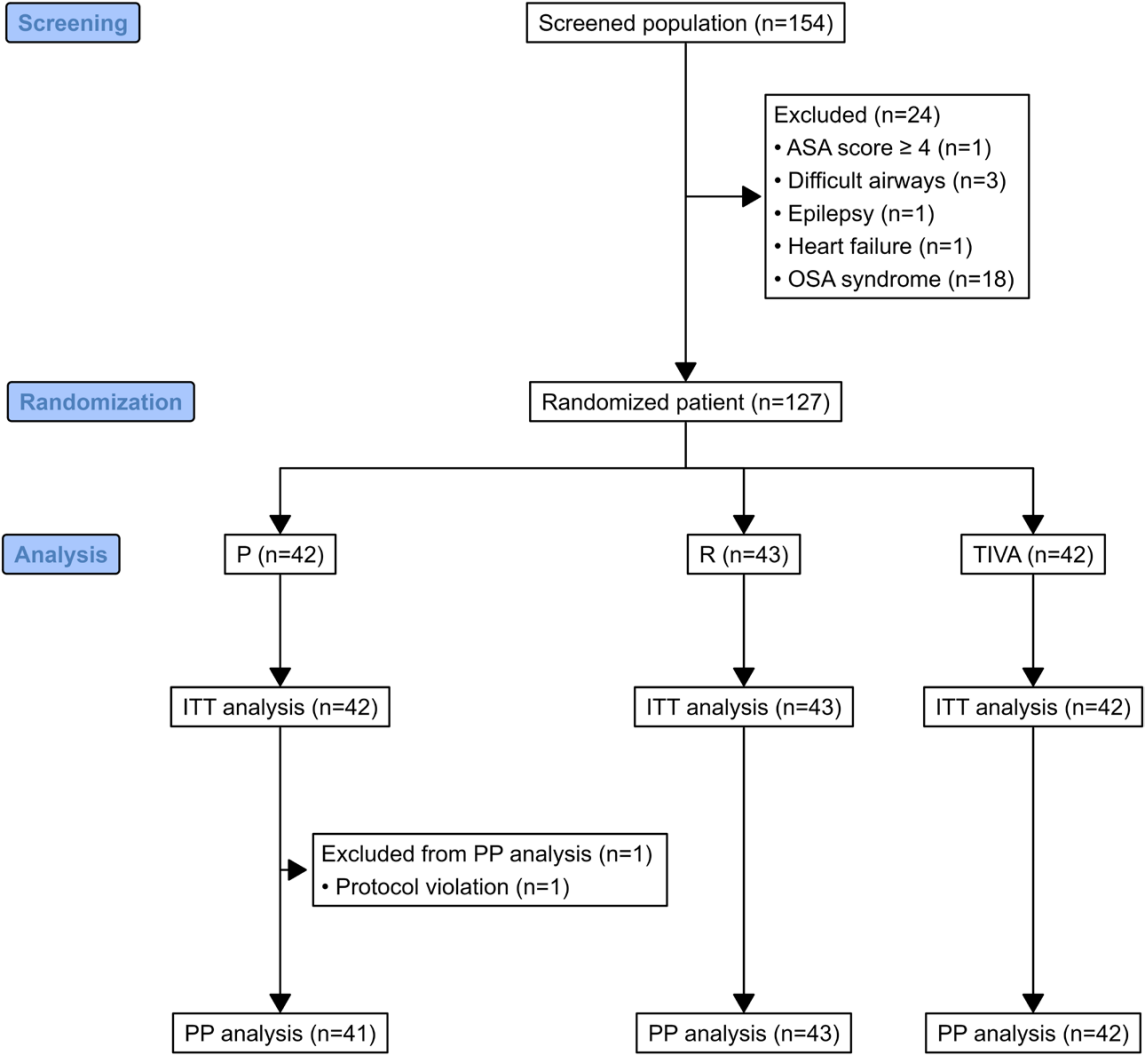
**Study flowchart.** ASA indicates American Society of Anesthesiologists; ITT, intention to treat; OSA, obstructive sleep apnea; PP per protocol; and TIVA, total intravenous anesthesia.

Between October 2023 and September 2024, 154 patients were assessed for eligibility, of whom 127 were randomly assigned to the P arm (n=42), R arm (n=43), or TIVA arm (n=42; Figure 1). The major exclusion criterion was the presence of obstructive sleep apnea syndrome. The mean age was 62.9 (SD 10.3); 54 (35.1%) were women, and 60 (47.2%) experienced paroxysmal AF. Other baseline characteristics were comparable among the groups (Table 1).

**Table 1.**
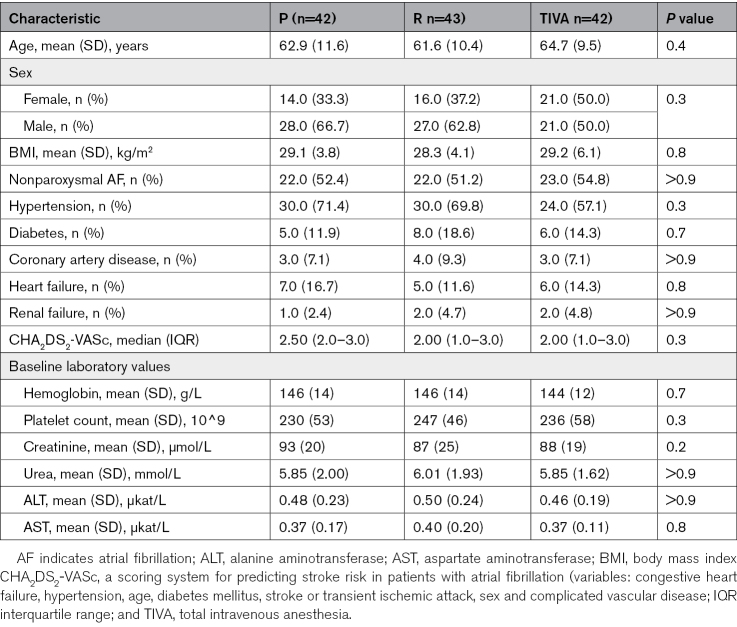
Baseline Characteristics

The median procedure duration for the total cohort was 58 minutes (IQR, 45–67). PVI was successfully achieved in all patients. For the non-pulmonary vein ablation lesions, a posterior wall ablation was performed in 65 (51.2%) patients, and a mitral isthmus ablation was performed in 60 (47.2%). The median number of PF pulses was 38 (IQR, 32–40) in patients with PVI only and 70 (IQR, 62–77) in patients with PVI combined with additional ablation lesions.

One patient in the P arm did not undergo PFA because of difficult vascular access and the risk of severe vascular complications and was excluded from the per-protocol analysis. Procedure characteristics and total sedation drug doses are detailed in Tables S3 and S4.

### Primary End Point

**Figure 2.**
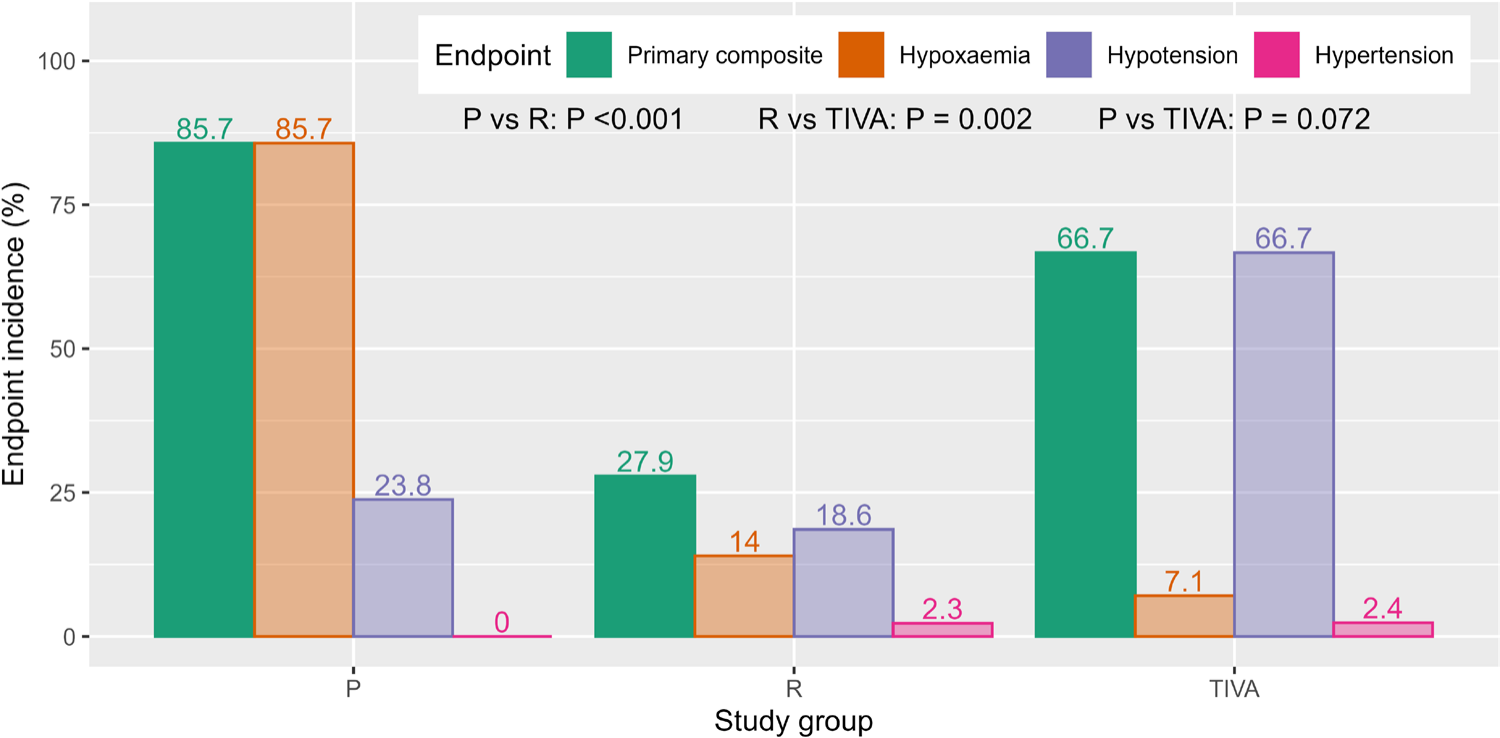
**Primary composite endpoint incidence.** TIVA indicates total intravenous anesthesia.

According to the intention-to-treat analysis, the incidence of the primary endpoint (P, 85.7%; R, 27.9%; TIVA, 66.7%) differed significantly among the study groups (*P*<0.001). The primary end point was significantly less frequent in arm R compared with arm P (27.9% versus 85.7%, *P*<0.001) and in arm R compared with arm TIVA (27.9% versus 66.7%, *P*=0.002). The event rate in the TIVA arm was not significantly different from that in the P arm (Table 2; Figure 2). In the per-protocol analysis, all pairwise comparisons were statistically significant (Table S5).

**Table 2.**
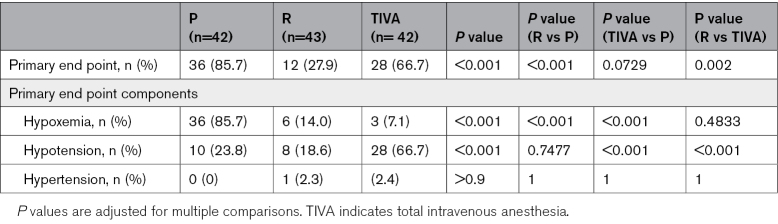
Primary End Point and Its Components

### Secondary Outcomes

#### Instability Events and Interventions

For components of the primary end point (ie, instability events requiring intervention), hypotension was more frequent in TIVA compared with the other arms (P versus TIVA: 23.8% versus. 66.7%, *P*<0.001; R versus TIVA: 18.6% versus 66.7%, *P*<0.001). Hypoxemia was most prevalent in arm P, with significantly fewer events in arms R and TIVA (P versus TIVA: 85.7% versus 7.1%, *P*<0.001; P versus. R: 85.7% versus. 14.0%, *P*<0.001). Hypertension was rare, with no significant differences among the study groups (Table S4).

Analyzing only instability events (oxygen saturation <85%, systolic blood pressure <85 mm Hg, or systolic blood pressure >200 mm Hg) irrespective of the need for intervention, we obtained similar results; hypoxemia was most frequent in the P arm (*P*<0.001), hypotension was most prevalent in the TIVA arm (*P*<0.001), and no significant difference was observed for hypertensive events. The P arm, which had the highest hypoxemia event rate, required more airway management interventions (ie, jaw thrust and increased fraction of inspired oxygen; *P*<0.001 for both). On the other hand, more patients in the TIVA arm required vasoactive drug administration (*P*<0.001) compared with the other 2 arms.

#### Total Procedure Time and BIS

Total procedure duration was similar across all arms (*P*=0.18); however, a trend toward a longer procedure time was observed in patients in the TIVA arm (Table S3).

**Figure 3.**
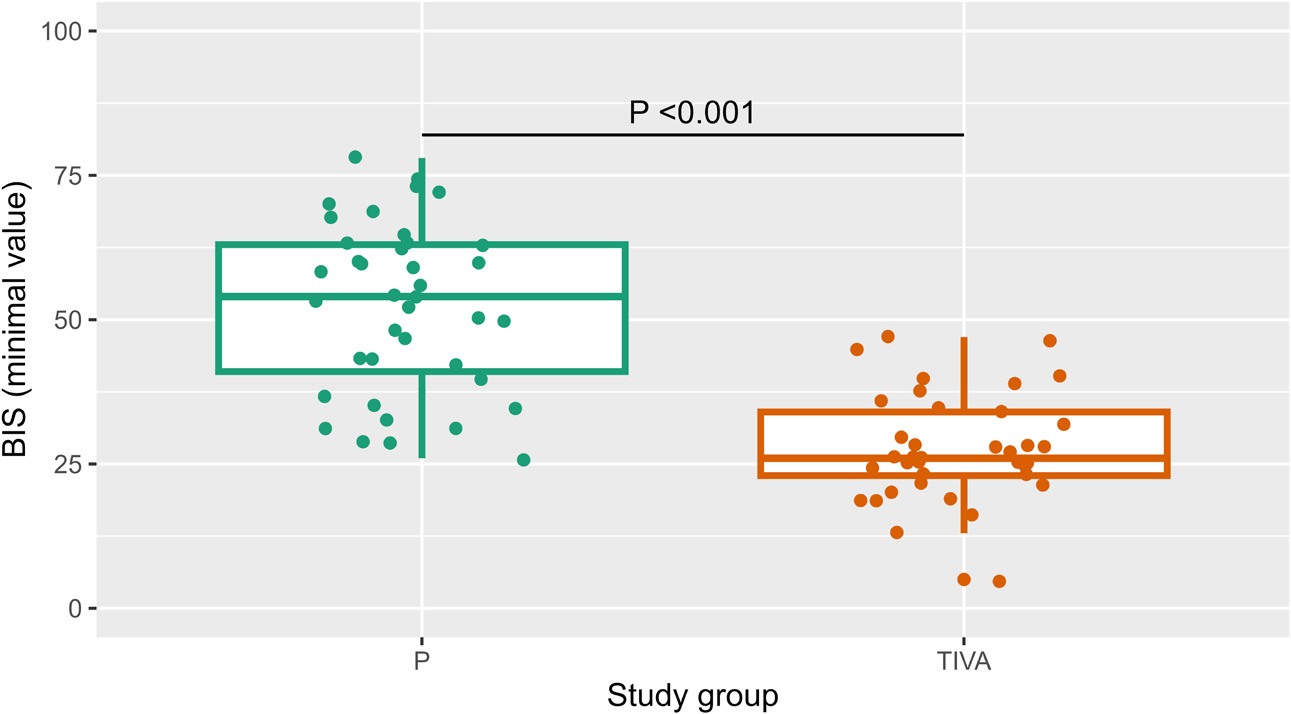
**Bispectral index (minimal value) by study arms (arm P and arm total intravenous anesthesia).** BIS indicates bispectral index.

The minimal BIS value was significantly lower (indicating deeper sedation) in the TIVA arm compared with the P arm (27.8 [SD 9.2] versus 52.2 [SD 14.4], *P*<0.001; Figure 3).

#### Patient, Operating Physician, and Anesthesiologist Satisfaction

**Figure 4.**
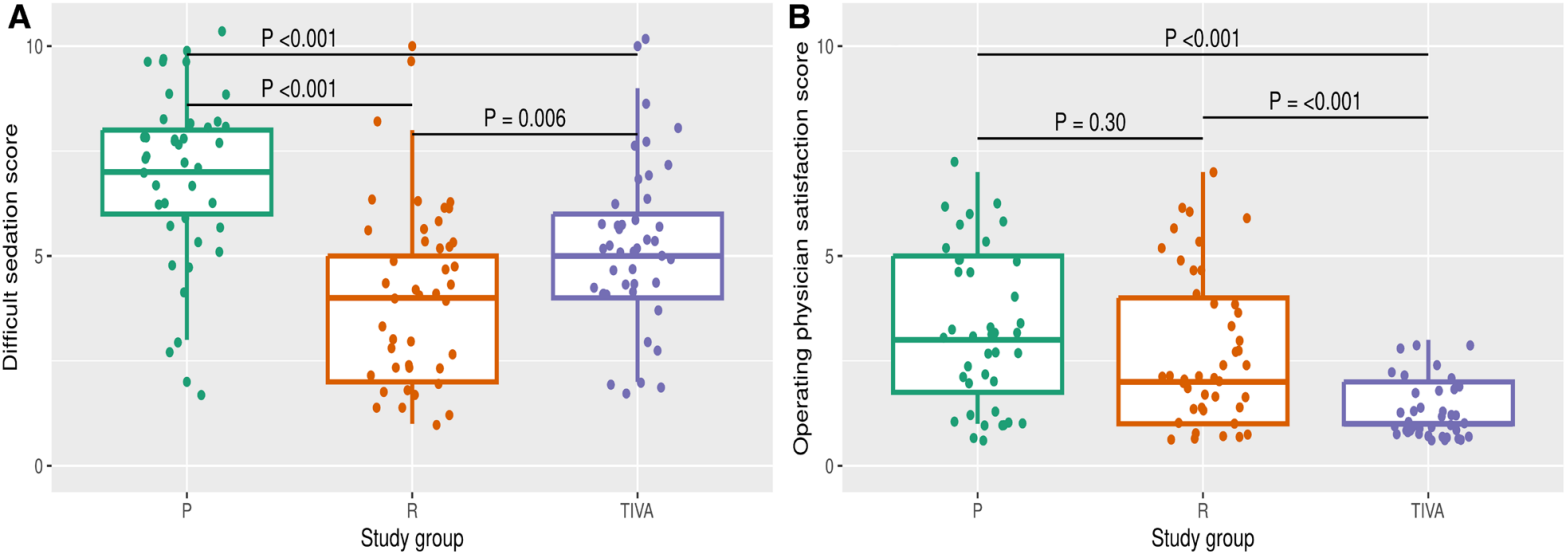
**Difficult sedation score and physician satisfaction score by study arms. A**, Difficult sedation score by study group (1–10 scale: 1 = easy; 10 = difficult). **B**, Operating physician satisfaction score by study group (1–10 scale: 1 = satisfied; 10 = not satisfied).

No significant differences in patient-reported satisfaction (PROcedural Sedation Assessment Survey) were found among the protocols. Based on procedure evaluations by the operating physicians, the best sedation quality was observed in the TIVA group. On the other hand, from the attending anesthesiologists’ point of view, patients in arm P were the most difficult to sedate (Figure 4).

#### End-Procedural PCO_*2*_, Serious Adverse Events, and Other Procedure-Related Adverse Events

End-procedural PCO_2_ levels were highest in the P arm and lowest in the TIVA arm, with significant differences among groups (*P*<0.001 for all comparisons).

Two serious adverse events occurred in the TIVA arm (one emergency intubation and one procedure discontinuation), but there was no significant difference in adverse event rates among groups.

In addition, 5 other procedure-related complications occurred (2 in arm P, 2 in arm R, and one in arm TIVA): one arteriovenous fistula, one pseudoaneurysm, one pharyngeal hematoma, one circumflex artery vasospasm, and one case of ketamine-related agitation. All complications were managed conservatively or spontaneously resolved, and no additional complications were reported after discharge (Table S2). For details of secondary end points, see Table S6.

## DISCUSSION

In this study, we compared 3 sedation approaches for the PFA procedure: (1) conventional DAS (arm P), intermittent boluses of propofol and opioids, commonly used in Europe without secured airways^20^; (2) optimized DAS (arm R), continuous infusion of remimazolam combined with ketamine boluses, aiming for stable sedation with fewer respiratory and hemodynamic side effects; and (3) TIVA (arm TIVA), continuous propofol infusion with opioid analgesia and secured airway (typically with an LMA), a method more prevalent in North America.^20^

We found that DAS based on continuous intravenous remimazolam with ketamine boluses led to significantly fewer adverse hypoxemia or hypotensive events compared with propofol-based DAS and TIVA. Although the overall primary outcome rate did not differ significantly between the P and TIVA arms, the nature of the adverse event varied; patients in the P arm primarily experienced periprocedural hypoxemia, whereas those in the TIVA arm had a higher incidence of hypotension. Although hypotension is common both in TIVA^12,21^ and during conscious sedation,^22^ and can be effectively managed, the 85% incidence of periprocedural hypoxia seen in our P arm was worrying. Hypoxia was accompanied by significant hypoventilation, as reflected in elevated end-procedure arterial pCO_2_. The P arm also had the lowest mean BIS value (52), which is within the GA range (40 to 60) rather than the expected analgosedation range (60 to 80).^23^ These events occurred despite excluding patients with sleep apnea, the presence of complete monitoring, and anesthesiologist oversight. In real-world settings, where nonairway-trained personnel often administer sedation, these risks could be even greater. In simple terms, we found that the depth of conventional analgosedation required for tolerance of PFA is that of GA, and, in turn, cannot be safely performed without securing the airway.

The novel remimazolam-ketamine regimen, which was originally described outside the context of cardiology,^24^ changes this. Remimazolam is a novel benzodiazepine with rapid clearance because of its metabolism by tissue esterases, making it significantly faster acting than midazolam.^25,26^ Compared with propofol, it causes fewer hypotensive episodes at GA induction^27^ and less respiratory depression.^12,22^ Ketamine, a fast-acting N-methyl-D-aspartate receptor antagonist, provides profound analgesia while preserving airway reflexes and only mildly affecting respiration.^28^ It also increases cardiac output and blood pressure via sympathetic activation.^29^ The remimazolam-ketamine combination offers several key advantages: (1) rapid onset and fast recovery, (2) stable hemodynamics, and (3) low risk of respiratory depression. Its main side effects, agitation and hallucinations, are mitigated by the use of benzodiazepines.^30,31^ The findings of our study reflect and confirm these benefits in the very specific context of PFA; these drugs allow enough flexibility to adjust the depth of anesthesia to the intensity needed for procedural stimulation, thus making PFA pulses tolerable while avoiding hypotension and reducing the loss of airway resulting from oversedation. Although the absolute rate of periprocedural hypoxia/hypotensive events (28%) was the lowest among all groups, it was still significant. Even with this optimized regimen, dedicated staff are still essential for managing analgosedation during PFA in electrophysiology labs, with backup from fully airway-trained personnel.

In many European countries, only anesthesiologists are permitted to administer propofol during analgosedation because of the risk of respiratory depression. However, in some centers, sedation for this procedure is administered by nonairway-trained personnel, which raises safety concerns; a global survey from 2010 to 2019^20^ reported that 28.6% of AF ablation centers performed deep sedation without an anesthesiologist on site, and 11.8% of respondents reported that an electrophysiologist could perform deep sedation independently and without additional personnel. Similarly, the recent EU-PORIA (EUropean Real World Outcomes with Pulsed Field AblatiOn in Patients with Symptomatic AtRIAl Fibrillation) registry,^7^ which analyzed 1233 PFA procedures conducted across 7 high-volume European centers, reported that 80% were performed with deep sedation and 20% with GA. Moreover, the ease of PFA and the steadily increasing number of PFA procedures will probably lead to an expanding pool of AF ablation candidates, including older, sicker patients with unrecognized coronary artery disease, for whom hypoxemia, combined with hypotension, could lead to significant clinical adverse events along with periprocedural complications. Even without acute and direct clinical consequences, hypoxemia can have delayed consequences, such as cognitive dysfunction,^32^ which was not measured in this trial but is nonetheless very important for patients. It is also worth noting that half of the clinical complications (adverse events) were associated with the analgosedation and not with the catheterization procedure itself.

Our trial results highlight the need to reconsider conventional sedation practices for PFA.

Procedure times and patient satisfaction were similar across all 3 arms. Surprisingly, TIVA with an LMA did not prolong procedures, as the time for GA induction was likely offset by smoother procedural progress (median difficulty score, 5 [IQR 4–6]; operator satisfaction score, 1 [IQR 1–2]). Operators rated sedation quality highest in the TIVA group, likely because of minimized involuntary movements. Although this difference in satisfaction was significant, it did not impact procedure duration. Notably, the procedures were performed using a single-shot catheter. However, when using a mapping system, patient movement could lead to electroanatomical map shifts, potentially extending procedure times. Anesthesiologists reported that sedating P arm patients was the most challenging, likely because of the higher incidence of primary end point events.

Finally, remimazolam-ketamine–based sedation offers promise and should be evaluated in randomized trials beyond the field of arrhythmology. Although most cardiology interventions require only mild sedation,^33^ more evidence is needed for procedures requiring DAS or GA, such as transcatheter aortic valve replacement. The largest randomized study on this topic, by Thiele et al, compared DAS and GA in 447 patients with transcatheter aortic valve replacement and found no difference in the primary composite end point (mortality, myocardial infarction, stroke, infection, and acute kidney injury at 30 days).^34^ However, inconsistencies in sedation protocols (38% propofol and 51% dexmedetomidine) complicated interpretation. Our study highlights that both sedation depth and drug choice matter, underscoring the need for further research using rigorous methodologies.

### Study Limitations

Several limitations of our study must be mentioned. First, this study was single center, and expertise levels may have varied among centers. However, standardizing the study protocol minimized this issue. Second, neither anesthesiologists nor procedural cardiologists were blinded to patient randomization, which could be a source of bias. In particular, the “difficult sedation” score should be interpreted with caution. Third, we excluded high-risk patients such as those with obstructive sleep apnea syndrome (or an apnea-hypopnea index ≥30), heart failure, or significant valvulopathies; this could limit the generalizability of our results. Fourth, the anesthetists were asked to strictly follow the study protocol. A physician highly experienced in sedation and anesthesia administration might have, in some cases, opted to modify the dosing or drugs based on the patient’s frailty and response to the study drugs. Fifth, we chose a composite primary end point; the clinical consequences of the components (hypotension, hypertension, and hypoxia) might, and likely do, differ. Last, although a multiple comparisons correction was applied in the analysis, the initial power analysis did not account for this correction; hence, the actual statistical power of our study could be lower than the planned 80%, increasing the risk of a type II error.

### Conclusions

In this prospective randomized controlled trial, which evaluated sedation regimens during pulsed-field catheter ablation of AF, we found that the remimazolam-ketamine DAS protocol led to significantly fewer clinically relevant adverse events than propofol-based DAS or TIVA. Regardless of the sedation regimen, we observed high rates of adverse events requiring immediate interventions, suggesting that a dedicated anesthesia provider needs to be present for safe conduction of the procedure.

## Article Information

### Acknowledgments

The authors thank the anesthesia assistants for their patience and willingness to consistently follow the study protocol. Recruitment and randomization would not have been possible without the help of Katerina Venclova, MD, who was essential in managing preanesthetic assessments. The authors also thank colleagues who provided anesthesia care when they were not available: Linda Thi Cao, MD; Petr Kafka, MD, PhD; Miroslav Keselica, MD; Martin Krbec, MD, PhD; Eva Matejkova, MD; Veronika Panska, MD; and Stepanka Vitovska, MD. Special thanks to Karol Curila, MD, PhD, for proofreading the article.

### Sources of Funding

This work was supported by the Charles University Research program “Cooperatio–Cardiovascular Science,” “Cooperatio–Intensive care Medicine,” and by the project National Institute for Research of Metabolic and Cardiovascular Diseases (CarDia), Program EXCELES, project ID LX22NPO5104. This work was also funded by the European Union – Next Generation EU.

### Disclosures

None.

### Supplemental Material

Supplemental Methods

Supplemental Tables S1–S6

Supplemental Figure S1
